# The Genetic Structure of *Phellinus noxius* and Dissemination Pattern of Brown Root Rot Disease in Taiwan

**DOI:** 10.1371/journal.pone.0139445

**Published:** 2015-10-20

**Authors:** Chia-Lin Chung, Shun-Yuan Huang, Yu-Ching Huang, Shean-Shong Tzean, Pao-Jen Ann, Jyh-Nong Tsai, Chin-Cheng Yang, Hsin-Han Lee, Tzu-Wei Huang, Hsin-Yu Huang, Tun-Tschu Chang, Hui-Lin Lee, Ruey-Fen Liou

**Affiliations:** 1 Department of Plant Pathology and Microbiology, National Taiwan University, No. 1, Sec. 4, Roosevelt Rd., Taipei City 10617, Taiwan; 2 Master Program for Plant Medicine, National Taiwan University, No. 1, Sec. 4, Roosevelt Rd., Taipei City 10617, Taiwan; 3 Plant Pathology Division, Taiwan Agricultural Research Institute, No. 189, Zhongzheng Rd., Wufeng Dist., Taichung City 41362, Taiwan; 4 Division of Forest Protection, Taiwan Forestry Research Institute, No. 53, Nanhai Rd., Zhongzheng Dist., Taipei City 10066, Taiwan; 5 Department of Crop Environment, Taitung District Agricultural Research and Extension Station, No. 675, Sec. 1, Zhonghua Rd., Taitung City 95055, Taiwan; Wuhan Botanical Garden of Chinese Academy of Sciences, CHINA

## Abstract

Since the 1990s, brown root rot caused by *Phellinus noxius* (Corner) Cunningham has become a major tree disease in Taiwan. This fungal pathogen can infect more than 200 hardwood and softwood tree species, causing gradual to fast decline of the trees. For effective control, we must determine how the pathogen is disseminated and how the new infection center of brown root rot is established. We performed Illumina sequencing and *de novo* assembly of a single basidiospore isolate Daxi42 and obtained a draft genome of ~40 Mb. By comparing the 12,217 simple sequence repeat (SSR) regions in Daxi42 with the low-coverage Illumina sequencing data for four additional *P*. *noxius* isolates, we identified 154 SSR regions with potential polymorphisms. A set of 13 polymorphic SSR markers were then developed and used to analyze 329 *P*. *noxius* isolates collected from 73 tree species from urban/agricultural areas in 14 cities/counties all around Taiwan from 1989 to 2012. The results revealed a high proportion (~98%) of distinct multilocus genotypes (MLGs) and that none of the 329 isolates were genome-wide homozygous, which supports a possible predominant outcrossing reproductive mode in *P*. *noxius*. The diverse MLGs exist as discrete patches, so brown root rot was most likely caused by multiple clones rather than a single predominant strain. The isolates collected from diseased trees near each other tend to have similar genotype(s), which indicates that *P*. *noxius* may spread to adjacent trees via root-to-root contact. Analyses based on Bayesian clustering, *F*_ST_ statistics, analysis of molecular variance, and isolation by distance all suggest a low degree of population differentiation and little to no barrier to gene flow throughout the *P*. *noxius* population in Taiwan. We discuss the involvement of basidiospore dispersal in disease dissemination.

## Introduction

Brown root rot caused by *Phellinus noxius* (Corner) Cunningham, a white rot fungus, occurs in tropical and subtropical areas worldwide. *P*. *noxius* is a member of the family Hymenochaetaceae, order Hymenochaetales, phylum Basidiomycota of the kingdom Fungi. When grown in potato dextrose agar (PDA), it forms colonies that are initially white to yellowish brown and with age are decorated with irregularly shaped lines or patches of dark brown tissues. Moreover, trichocysts and arthospores are formed in culture, although no clamp connection is observed [1]. Formation of arthospores in culture is rarely seen in other species of *Phellinus*, so it may serve as a reference for the identification of *P*. *noxius*. However, when grown on sawdust medium, *P*. *noxius* can form thin-layered, flat basidiocarps that are initially yellowish brown and later turn brown and dark gray, similar to those found in nature [2].

To date, more than 200 agricultural and forest plant species have been reported as hosts of *P*. *noxius*; most are woody but some are herbaceous plant hosts [1]. As revealed by cross-inoculation studies, *P*. *noxius* does not exhibit host specificity, although varying degrees of resistance were detected in different hosts [3–5]. Infection by the fungus usually begins in the roots and spreads to the collar. *P*. *noxius* can colonize both lateral and tap roots. The interior root tissue turns brown at first and then white and soft, with a network of dark brown lines all over. Moreover, the roots and stem bases of infected trees are covered with dark brown mycelial mats of *P*. *noxius* [6]. As the disease progresses, the trees show symptoms of chlorosis and defoliation owing to the fungus impairing root functions. Aboveground symptoms are usually not noticed until after extensive root damage. Eventually, the trees may die, often because they topple over in strong winds. In some cases, infection with *P*. *noxius* results in quick decline of trees, causing death within a few months of infection [1]. Basidiospores of *P*. *noxius* can cause brown root rot in newly generated tree stumps, followed by movement of the fungus from the stumps to roots [7]. Stumps and roots infected with *P*. *noxius* can serve as viable inoculum sources for years [7,8]. In addition, the fungus may be transferred from the infected to adjacent healthy trees through points of root contact [4]. The disease may introduce to new areas with transfer of the seedlings infected by *P*. *noxius* in the nursery. However, arthospores produced by *P*. *noxius* are not considered an important source of inoculum because they are absent in the field [7].

In Taiwan, brown root rot caused by *P*. *noxius* was first reported by Sawada in 1928, but at that time the fungus was identified as *Fomes lamaensis* [9]. Later, it was considered a synonym of *Fomes noxius* [10] and now as *P*. *noxius* [2,11]. Despite these early reports of the occurrence of brown root rot, the disease has not been a major concern and was neglected for a long time in Taiwan. The situation changed in the 1990s, when many cases of brown root rot were reported. The damage it caused to fruit trees such as longan (*Dimocarpus longana* = *Euphorbia longana*), litchi (*Litchi chinensis*), sugarapple (*Annona squamosa*), plum (*Prunus mume*), and loquat (*Eriobotrya japonica*) as well as some old trees of cultural and historical significance, including camphor (*Cinnamomum camphora*) and Taiwan Banyan (*Ficus macrocarpa*), have drawn great concern from the public [1,2,6]. Brown root rot caused by *P*. *noxius* has become an enormous threat to numerous woody fruit and ornamental trees all over Taiwan. For effective control of the disease, we must understand the genetic structure and dissemination pattern of *P*. *noxius*.

Previously, genotypes of *P*. *noxius* isolates have been analyzed on the basis of somatic compatibility, esterase isozyme patterns, and sequences of the ribosomal internal transcribed spacer [12,13]. However, these markers have drawbacks in terms of low polymorphism and sometimes poor reproducibility. Microsatellites, also known as simple sequence repeats (SSRs), are fragments of DNA composed of repeating motifs of 2- to 6-bp nucleotides arranged in tandem. Individuals may differ in the number of repeats because of relatively high rates of errors occurring in SSR regions during DNA replication (slippage) and recombination (unequal crossover). With the advantages of high reproducibility, high polymorphism, and codominance in inheritance, SSR markers have gained considerable importance in recent years and have been widely used in the study of population structure of fungal and oomycete pathogens [14–17].

In this study, we developed a SSR genotyping system and used it for the analysis of *P*. *noxius* isolates from different areas of Taiwan. We investigated population structure, genetic diversity, and distribution of genetic variation in *P*. *noxius* with respect to geographic location and host. We found that no dominant strain of *P*. *noxius* exists in the field. Investigations of population diversity and differentiation throughout Taiwan (island-wide scale) and in a small urban area of 3 x 1.2 km^2^ in Taipei (local scale) indicated that brown root rot may disseminate over short distances via root-to-root contact of hosts, and genetically variable basidiospores are likely responsible for the long-distance dispersal.

## Materials and Methods

### Collection, isolation, and culture of *P*. *noxius*

To isolate *P*. *noxius*, the infected plant tissues were excised into small pieces, surface-sterilized in 0.5% NaOCl for 1 min, and cultured on a selective medium [malt extract agar (MEA) or potato dextrose agar (PDA) containing 100 ppm streptomycin sulfate, 10 ppm benomyl, and 10 ppm dichloran] [18] at room temperature for several days. A single hyphal tip or single arthospore of *P*. *noxius* was then transferred to a new PDA plate and cultured for further use. The identity of the fungus was verified by morphological characteristics and analysis by PCR with two sets of species-specific primers, PN-1F/PN-2R [19] and G1F/G1R [20]. In total, we isolated 329 *P*. *noxius* samples from 73 tree species belonging to 34 different families (S1 Table). Most of the isolates were collected from Moraceae, Leguminosae (Fabaceae), Sapindaceae, and Lauraceae.

### Nuclear quantification

To observe nuclei present in the growing hyphal tips, an agar block excised from the actively growing margin of the *P*. *noxius* colony was put on a sterilized slide in a sterile moist chamber and incubated for 2 days. After removal of the agar block, the mycelium present on the slide was mounted in 20 μl of a DAPI (4',6-diamidino-2-phenylindole) solution (10 μg/ml in ddH_2_O) for 10 min and then sealed with a coverslip. DAPI staining was also conducted to observe the conidia from a naturally occurring basidiocarp and the arthospores dislodged from the *P*. *noxius* colony. Material was observed under a Leica DMLB microscope (Buffalo Grove, IL, USA) equipped with filter cube A (BP 340–380 nm, LP 425 nm). Images were captured by using a Canon (Ohta-ku, Tokyo, Japan) digital camera EOS 550D.

### DNA extraction

For each *P*. *noxius* isolate, the mycelium was taken from 7- to 10-day-old culture actively grown on the MEA medium. The mycelium was ground in liquid nitrogen by using a mortar and pestle or homogenized by using an SH-100 sample homogenizer (Kurabo, Osaka, Japan). About 50 mg of pulverized genomic DNA was extracted following a standard CTAB extraction protocol [21], with 750 μl CTAB extraction buffer [2% (w/v) hexadecyltrimethylammonium bromide, 1.4 M NaCl, 20 mM EDTA (pH 8.0), 0.1 M Tris-HCl (pH 8.0), 2% PVP, 0.2% (v/v) 2-mercaptoethanol], 750 μl PCI (phenol: chloroform: isoamyl alcohol = 25:24:1, v/v/v), and 450 μl isopropanol. The phenol-chloroform extraction and ethanol precipitation of DNA were performed according to standard procedures.

### SSR marker development

Polymorphic SSR markers were developed by a combined analysis based on *P*. *noxius* sequences obtained by next-generation sequencing (NGS). Genomic DNA libraries for five *P*. *noxius* isolates, with an average insert size of 200 bp, were prepared and sequenced as 2 × 101 bp paired-end reads on an Illumina HiSeq 2000 at Yourgene Bioscience (Taipei, Taiwan). The five *P*. *noxius* isolates included a single-basidiospore isolate, Daxi42 from Yilan city in Taiwan, and four additional isolates including I172B from Yilan city in Taiwan, K231-12A from Kaohsiung city in Taiwan, B109B from Sarahama, Miyako Island in Japan, and B95A from Pekanbaru in Indonesia. A mate-paired library (insert size: 2 kb) of Daxi42 was also sequenced as 2 × 101 bp paired-end reads. Low quality bases (error probability < 0.05, Q13) and contaminated-Illumina adapter sequences were trimmed. Reads < 35 bp long were also discarded. With the data generated from paired-end and mate-paired sequencing, a draft genome assembly of Daxi42 was created from the clean and filtered reads *de novo* assembled by using Velvet [22]. Repeating patterns of 2 to 5 bp, with lengths ≥ 10 bp, in the assembled contigs were identified by using the Sputnik program [23]. To further identify the SSR regions of potential polymorphism, we extracted the filtered reads containing repeating patterns from the isolates I172B, K231-12A, B109B, and B95A, respectively, then mapped those reads to the contigs of Daxi42. Each potential region was checked manually by multiple alignment analysis with ClustalX [24]. The primers for detecting SSR polymorphisms were designed by using Primer3 v0.4.0 [25], based on the conserved sequences flanking the SSR motifs.

### SSR genotyping

Genotypic analysis followed a modified fluorescence-based SSR genotyping method [26]. To minimize the inaccuracy of genotyping due to non-templated nucleotide addition by *Taq* DNA polymerase, we added an extra sequence to the 5’ end of each primers (PIGtailing): 5'-ACGACGTTGTAAAA for the forward primer and 5'-GTTTCTTTTCCCATTA for the reverse primer [27]. Each PCR reaction was performed in 10-μl reaction mixture containing 20 to 50 ng genomic DNA, 0.2 mM dNTP, 1X ImmoBuffer [16 mM (NH_4_)_2_SO_2_, 0.01% Tween-20, 100 mM Tris-HCl, pH 8.3] (Bioline, London, UK), 2 mM MgCl_2_, 40 nM locus-specific SSR primers, 80 nM fluorescence dye-labeled *tagF* primer (5’- VIC/FAM/NED/PET-ACGACGTTGTAAAA), 80 nM unlabeled *tagR* primer (5’- GTTTCTTTTCCCATTA), and 0.25 U Immolase DNA polymerase (Bioline). The thermal cycling parameters were 1 cycle of 95°C for 10 min, 20 cycles of 92°C for 30 s, 63°C for 90 s, and 72°C for 60 s, followed by 40 cycles of 92°C for 15 s, 54°C for 30 s, and 72°C for 60 s, and a final extension step of 72°C for 30 min. Amplicons labeled with different fluorescent dyes were multiplexed (VIC:FAM:NED:PET = 2:4:4:6), mixed with formamide and GeneScan-500 LIZ size standard (Applied Biosystems, Foster City, CA, USA), and analyzed on the Applied BioSystems 3730xl DNA Analyzer at Genomics BioSci & Tech. (New Taipei City, Taiwan). The sizes of amplicons were scored by using GeneMapper v3.0 (Applied Biosystems).

### Data analysis

A total of 329 isolates were genotyped for the 14 newly-developed SSR markers. For each of the 14 SSR loci, number of alleles, major allele frequency, number of genotypes, genetic diversity, heterozygosity, and polymorphism information content (PIC) were analyzed by using PowerMarker v3.25 [28]. Before genetic analyses, linkage disequilibrium between pairs of markers was tested with FSTAT v2.9.3.2 [29]. The significance level was adjusted by Bonferroni correction for multiple comparisons.

The Bayesian genetic clustering analyses were implemented in STRUCTURE 2.3.4 [30] to determine the most probable number of clusters (*K*). Individual genotypic data were used to evaluate models assuming different numbers of genetic clusters (*K* = 1–20 for the overall population and *K* = 1–10 for the subpopulation in Taipei) based on the posterior probabilities. Under the assumptions of admixture and correlated allele frequencies, the program was run with each *K*-value replicated 10 times, with the burn-in period and Markov Chain Monte Carlo (MCMC) lengths both set to 500,000. Appropriate number of *K* was estimated following the Δ*K* method by Evanno et al. [31]. For the Taipei subpopulation, discriminant analysis of principal components (DAPC) was implemented with the adegenet package [32] (function dapc) in R [33]. The prior information regarding grouping was defined based on the best *K* inferred from the STRUCTURE analysis.

**Fig 1:**
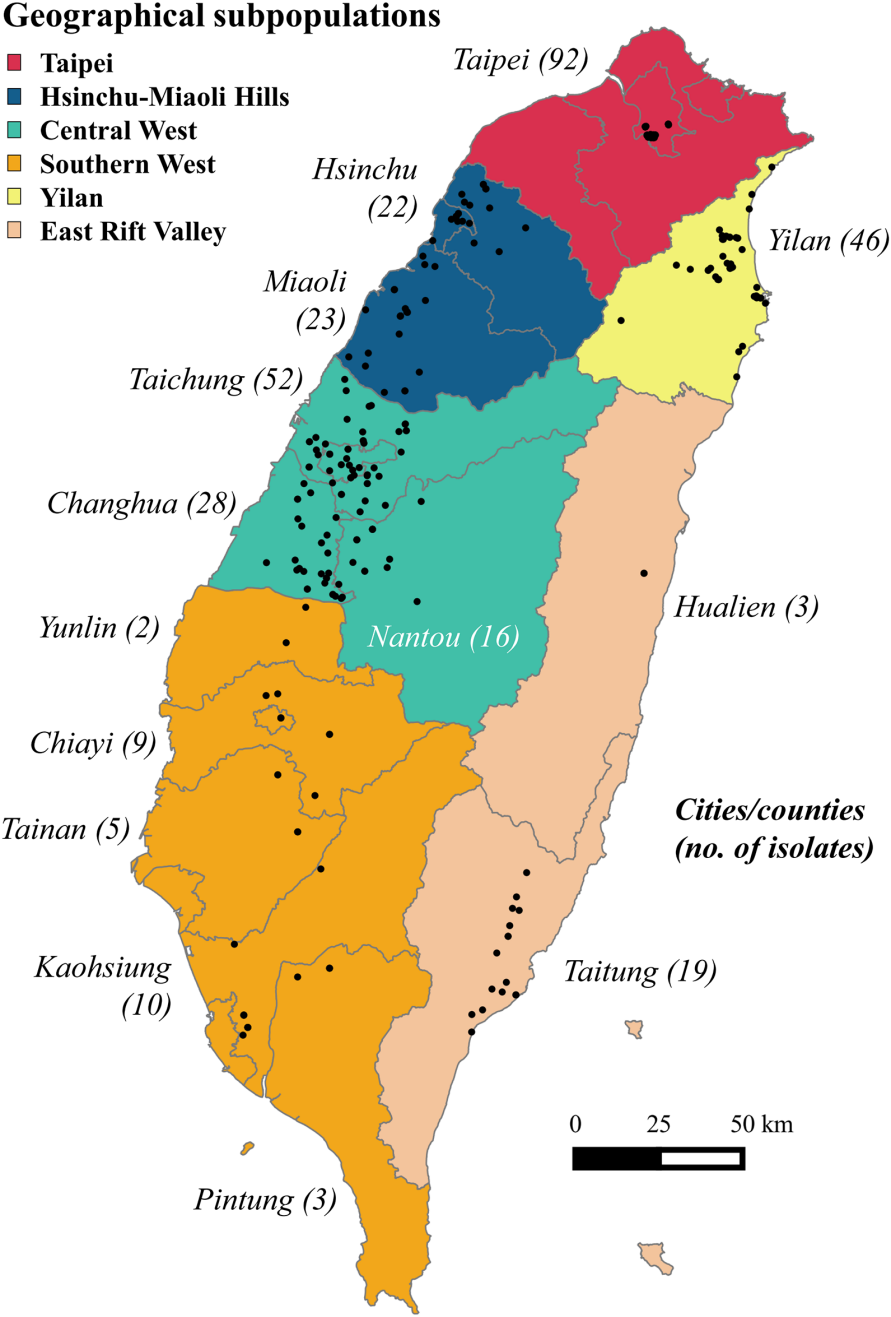
A total of 3291 isolates of *Phellinus noxius* were sampled from 14 cities/counties in Taiwan. The cities/counties and the numbers of *P*. *noxius* isolates from the areas are in italic. Different colors indicate the extent of the six geographical subpopulations: Taipei (TP), Hsinchu-Miaoli Hills (HM, including Hsinchu and Miaoli), Central West (CW, including Taichung, Changhua, and Nantou), Southern West (SW, including Yulin, Chiayi, Tainan, Kaohsiung, and Pintung), Yilan (YL), and East Rift Valley (EV, including Hualien and Taitung).

Since the STRUCTURE analysis did not detect significant clustering structure for the 329 *P*. *noxius* isolates (details in Results), we arbitrarily divided the whole population into six geographical subpopulations: Taipei (TP), Hsinchu-Miaoli Hills (HM), Central West (CW), Southern West (SW), Yilan (YL), and East Rift Valley (EV) (Fig 1). To assess the genetic diversity within the entire population in Taiwan and the six geographical subpopulations, mean number of alleles per locus (*N*_A_), allelic richness averaged over loci (*A*_R_) (rarefaction-based estimation), observed heterozygosity (*H*_O_), and unbiased expected heterozygosity (*H*_S_) [34] were calculated with FSTAT v2.9.3.2 [29]. To analyze clonality, distinct multilocus genotypes (MLGs) were identified by using GenClone 2.0 (http://www.ccmar.ualg.pt/maree/software.php?soft=genclon) [35].

The degree of genetic differentiation was evaluated by Wright’s *F* statistics [36]. Global *F*_ST_ values across all the subpopulations and pairwise *F*_ST_ values between pairs of subpopulations were calculated with FSTAT v2.9.3.2 [29,37], with the significance levels determined with 1000 permutations and adjusted by Bonferroni correction for multiple tests. For the overall population and subpopulations, the fixation indices (*F*_IS_) were calculated with FSTAT v2.9.3.2 [29] and the deviations from Hardy—Weinberg equilibrium (HWE) for each locus were estimated with Genepop 4.2 [37]. Deviation from HWE was tested by the probability test, with unbiased *P* values estimated by a Markov chain method (10000 dememorization steps, 100 batches, and 5000 iterations per batch). To evaluate the proportions of genetic variance explained by the effects of geographical subpopulations, families of host plants, and collection years, we used AMOVA [38] performed with ARLEQUIN v3.5.1.2 [39]. The test for collection years was run on the basis of 18 individual years (1989–2012) or three time periods (1989–1999, 2001–2009, 2010–2012). AMOVA was also conducted to test the within- and between-clusters variation in the Taipei subpopulation.

Analysis of isolation by distance between pairs of individuals was performed as described Rousset [40] with Genepop 4.2 [37]. The significance of correlation was evaluated with a Mantel’s test [41] with 1000 permutations in the ISOLDE program implemented in Genepop 4.2. The geographic distances were estimated on the basis of the global positioning system (GPS) coordinates of the collection sites. TWD97 Transverse Mercator 2° zone 121 X—Y coordinate system was used.

SSR genotypes of the 329 isolates were used to construct a phylogenetic dendrogram based on simple matching coefficient by the neighbor-joining (NJ) method with DARwin 6.0.5 [42]. Bootstrap analysis with 2000 iterations was performed to determine the reproducibility of the dendrogram.

## Results

### Marker development and SSR loci considered for analyses

To obtain nucleotide sequences of *P*. *noxius*, we performed Illumina sequencing and *de novo* assembly with DNA from a single-basidiospore isolate, Daxi42. We also generated low-coverage sequencing data for four other *P*. *noxius* isolates from Taiwan, Japan, and Indonesia (I172B, K231-12A, B109B, and B95A). After quality trimming, we obtained approximately 25 Gb paired-end and 164 Mb mate-paired sequences for Daxi42 and approximately 2 Gb paired-end sequences for each of the other four isolates. The assembly resulted in 15,966 contigs with a N50 scaffold length of 47,281 bp, and the size of the assembled genome was 39,988,869 bp. A total of 12,217 SSR regions were identified in the Daxi42 genome, including repeat regions of 1,653 dinucleotide, 4,012 trinucleotide, 3,126 tetranucleotide, and 3,426 pentanucleotide. By comparing Daxi42 SSRs with nucleotide sequences of the other four isolates, 154 SSR regions with potential polymorphisms were identified, from which 76 SSR primer sets were designed. To validate the applicability of these markers, we analyzed the corresponding SSR loci for eight *P*. *noxius* isolates collected from different areas of Taiwan. To verify that these markers were locus-specific and the observed differences in lengths were allelic variations, we sequenced the amplicons of two dominant and two rare alleles for each marker by the dideoxy termination method. Based on the PCR amplification efficiency, reproducibility, and discriminating capacity (> 2 bp difference between alleles), a set of 14 SSR markers were chosen and used for subsequent analyses (Table 1).

**Table 1:** The 14 SSR primers developed in this study.

SSR locus	Product size (bp)	Motif	Forward primer ^a^	Reverse primer ^a^
02	188–232	(GAA)_4_	ACGACGTTGTAAAAGCAATTAGACGGGGAGAGGA	GTTTCTTTTCCCATTACTGCCGTAGACATCCCCATT
05	218–254	(TGA)_6_	ACGACGTTGTAAAACCTGAACGAAAGGATGCAGTT	GTTTCTTTTCCCATTAGGTGGCTCGTTGTCAGGTATG
07	132–150	(ATG)_6_	ACGACGTTGTAAAATCGCCGACAACAGTAACGAC	GTTTCTTTTCCCATTAGCACGACCAACACCATCATT
08	360–375	(AAG)_6_	ACGACGTTGTAAAAGCAGACGAAATTGCCTTTCA	GTTTCTTTTCCCATTACGGCGGGTATATTCTGTGGT
12	288–330	(CGA)_5_	ACGACGTTGTAAAATCGAGTCGGATCACTTCGTC	GTTTCTTTTCCCATTAAGGAGCAGATGGATGGTGGT
16	261–320	(AG)_16_	ACGACGTTGTAAAATGGTGAGGAGGATGGAAAGG	GTTTCTTTTCCCATTATTGATTGAACAAGTGAACAACAGC
19	326–361	(CA)_12_	ACGACGTTGTAAAAGACGCCTTTATCGTTTGAGGA	GTTTCTTTTCCCATTAGGGATAGACACCGCTGAAGG
27	308–342	(ATG)_11_	ACGACGTTGTAAAATTTGGACAGGACGAGCTTGA	GTTTCTTTTCCCATTATCCAAGGCCGCTTTACTCAT
28	205–238	(ATA)_6_	ACGACGTTGTAAAACCTGATTTGGATGCGGGTAT	GTTTCTTTTCCCATTACCACCAGTCCCACCGATAAT
29	228–268	(ACA)_9_	ACGACGTTGTAAAATACCCCAGTCCCGTCAAAAC	GTTTCTTTTCCCATTAAGCGAGAGTGCCTCCTGTTC
31	147–226	(AT)_11_	ACGACGTTGTAAAATGTACGTGTCCCAGATCCTGA	GTTTCTTTTCCCATTATCAAATGAGGGCGGGATATT
51	196–257	(AT)_7_	ACGACGTTGTAAAAGCTTTTGGTCAACGGTTTCG	GTTTCTTTTCCCATTATGACCATAGAAAGCCCCATGT
58	180–227	(ACA)_12_	ACGACGTTGTAAAAGACGTTAACGGCAACCAGAA	GTTTCTTTTCCCATTAAGCGTTAGCACCACCGTTA
61	304–332	(TGC)_7_	ACGACGTTGTAAAATGTTGGCCTGGTTGATTCTG	GTTTCTTTTCCCATTAGGCACAAACACCGCAAATAA

^a^ For genotyping by multiplex-ready PCR, a PIG-tail tag sequence was added to the 5’ end of each SSR primer: 5'-ACGACGTTGTAAAA for the forward primer and 5'-GTTTCTTTTCCCATTA for the reverse primer.

Our preliminary screening revealed that 11 out of the 14 markers identified more than two putative alleles (3–4 alleles, mostly 3 alleles) in some individual isolates (Table 2 and S2 Table). In total, 45, 6, and 7 isolates were found to carry more than two alleles at 1, 2, and 3 SSR loci, respectively. To confirm the identity of these “multi-allelic” fragments in the amplicon, we conducted a progeny test for the isolate HC2010MR03, which displayed 4 distinct alleles for the SSR61 locus. Briefly, 16 single-arthospore progeny isolates were generated and genotyped for SSR61. Segregation of the allelic fragments was observed in the progeny population, with each progeny isolate inheriting 2–4 alleles of the marker (2, 3, and 4 alleles detected in 13, 2 and 1 isolates, respectively). Combined with the observation of various numbers of nuclei in the hyphae and arthospores of *P*. *noxius* (S1 Fig), we speculate that *P*. *noxius* has multinucleate cells containing genetically different nuclei. With this in mind, we used analytical approaches developed for diploids in this study, because current knowledge of sexual and asexual reproduction of *P*. *noxius* is quite limited, and challenges in population genetics of heterokaryotes and polyploids remain [43]. Therefore, for each of the 14 SSR markers, only the two alleles with the first and second greatest fluorescence intensity were used to represent the alleles dominant in an individual. (In most of the multi-allelic cases we observed, the fluorescence intensity was more than five-fold greater for these two alleles than the rest of the alleles, which indicates that they are representative allele samples.)

**Table 2:** Summary statistics of the 14 SSR markers utilized to assess the overall 329 isolates of *Phellinus noxius* in Taiwan.

SSR locus	Sample size	No. of isolates observed	No. of multi-allelic isolates observed^b^	No. of alleles	Major allele (amplicon size, bp)	Major allele frequency	No. of genotypes	Gene diversity	Heterozygosity	PIC^c^
02	329	324	9	8	194	0.31	16	0.74	0.52	0.69
05 ^a^	329	324	0	4	218	0.95	6	0.10	0.09	0.10
07	329	327	2	7	144	0.72	9	0.41	0.27	0.35
08	329	317	7	7	363	0.40	17	0.69	0.36	0.64
12	329	320	0	6	291	0.87	9	0.24	0.18	0.23
16	329	320	1	11	316	0.79	18	0.37	0.21	0.35
19	329	315	17	16	326	0.27	55	0.86	0.46	0.84
27	329	320	12	13	321	0.19	33	0.84	0.52	0.82
28	329	327	4	12	226	0.58	28	0.58	0.24	0.52
29	329	326	5	9	237	0.52	19	0.64	0.37	0.59
31	329	323	1	10	152	0.72	18	0.46	0.34	0.44
51	329	312	2	10	222	0.75	19	0.42	0.15	0.40
58	329	322	0	9	180	0.89	16	0.21	0.13	0.21
61	329	323	18	9	316	0.44	20	0.71	0.42	0.67

^a^ SSR05 was not considered in the subsequent analyses because of its low polymorphism and the significant linkage disequilibrium with SSR16.

^b^ Multi-allelic isolate: the isolate carrying more than two alleles for the SSR locus.

^c^ PIC: polymorphism information content

Analysis of 329 *P*. *noxius* isolates sampled across all of Taiwan revealed the presence of 131 alleles detected by the 14 SSR markers, ranging from 4 alleles at the SSR05 locus to 16 alleles at the SSR19 locus (Table 2). The mean PIC value was 0.489 (range 0.10–0.84). Significant linkage disequilibrium (Bonferroni-corrected *P* = 0.00055) was observed between SSR05 and SSR16, as well as between SSR27 and five other loci (SSR02, SSR19, SSR29, SSR31, and SSR61). The SSR05 locus was excluded from subsequent analyses because it was less informative than SSR16 and the rest of the markers (Table 2; the PIC values for SSR05 and SSR16 were 0.1 and 0.35, respectively; the major allele frequency for SSR05 was 0.95). The SSR27 locus was highly polymorphic and informative (Table 2; genetic diversity = 0.84, PIC = 0.82). To determine whether the inclusion of this marker would lead to biased results and interpretations, we performed genetic analyses for the overall population and the six geographical subpopulations of *P*. *noxius* using the datasets with and without SSR27. The use of SSR27 did not affect the levels of genetic diversity, fixation index, and genetic differentiation. Because SSR27 would provide useful information, we retained it in the study.

### Population diversity

The computed values of *N*_A_, *A*_R_, *H*_O_, *H*_S_, *F*_IS_ and MLGs are in Table 3. Across the 13 SSR markers included in the analyses (SSR05 excluded), a total of 127 alleles were detected (Table 2). The mean number of alleles per locus (*N*_A_) and average allelic richness (*A*_R_) values were 9.77 and 5.285 in the overall population, and 4.69–7.62 and 4.619–5.379 in the six geographical subpopulations. Within all the sampled 329 *P*. *noxius* isolates and isolates in the subpopulations, the mean unbiased heterozygosity (*H*_S_) ranged from 0.524 to 0.558 and the observed heterozygosity (*H*_O_) from 0.275 to 0.372. Heterozygosity deficit and significant deviations from Hardy-Weinberg expectations (*P* < 0.001) were observed (overall: *F*_IS_ = 0.41; subpopulations: *F*_IS_ = 0.311–0.474). Tests for Hardy-Weinberg expectation were also conducted for each SSR locus for the overall population, and a significant departure (*P* = 0) was detected for all the 13 SSR loci. Among the 265 *P*. *noxius* isolates successfully genotyped for the 13 SSR loci (~20% of the isolates had a few missing data), 259 MLGs were identified, so most individuals (~98%) showed unique genotypes. In summary, for each of the abovementioned statistics, similar results were obtained for the overall population and the six subpopulations, which suggested comparable level of genetic diversity among *P*. *noxius* isolates collected from different areas of Taiwan.

**Table 3:** Summary statistics for the genetic diversity of the overall population and the six geographical subpopulations of *Phellinus noxius*. *N*_A_: mean number of alleles per locus; *A*_R_: allelic richness averaged over loci; *H*_O_: observed heterozygosity; *H*_S_: unbiased expected heterozygosity; *F*_IS_: fixation index; *F*_ST_: differentiation index; MLG: multilocus genotype.

Population^a^	No. of Isolates	*N*_A_	*A*_R_	*H*_O_	*H*_S_	*F*_IS_	*F*_ST_	MLG^b^
**TP**	92	7.62	5.119	0.328	0.554	0.408	0.003	69/75 (0.92)
**HM**	45	5.85	4.883	0.335	0.526	0.364	0.018	40/40 (1.00)
**CW**	96	7.08	5.379	0.302	0.558	0.459	0.014	74/74 (1.00)
**SW**	29	5.00	4.750	0.308	0.538	0.428	0.051	24/24 (1.00)
**YL**	45	5.54	4.829	0.372	0.54	0.311	0.008	36/36 (1.00)
**EV**	22	4.69	4.619	0.275	0.524	0.474	0.063	16/16 (1.00)
**Overall**	329	9.77	5.285	0.313	0.538	0.410	0.015	259/265 (0.98)

^a^ Overall: entire Taiwan; TP: Taipei; HM: Hsinchu-Miaoli Hills (HM); CW: Central West; SW: Southern West: YL: Yilan; EV: East Rift Valley.

^b^ The number of distinct MLGs/ the number of isolates used for MLG analysis. The value in the parenthesis represents the proportion of distinct MLGs in the population.

### Population structure and differentiation

**Fig 2:**
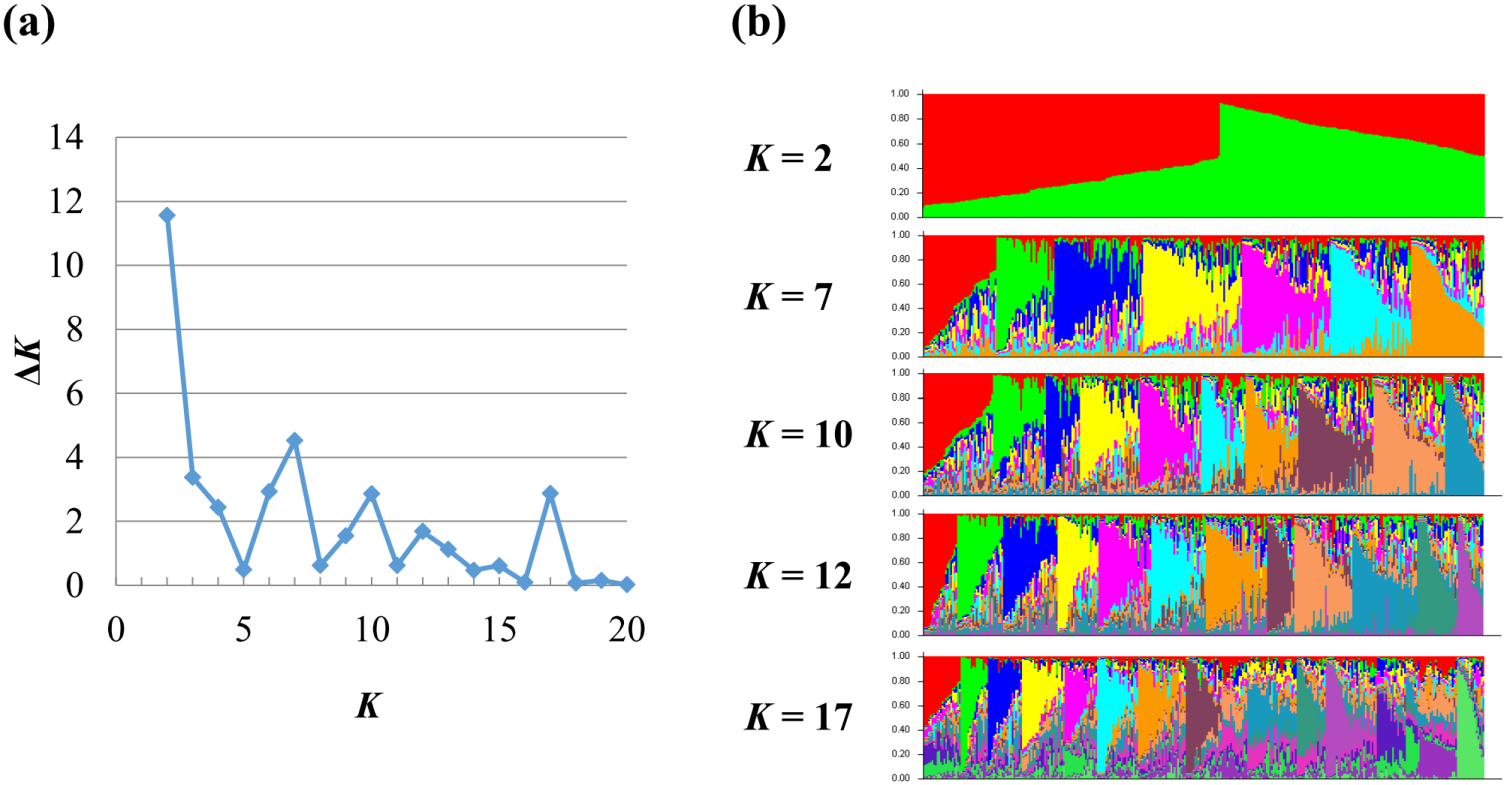
Estimated *Phellinus noxius* population structure in Taiwan by Bayesian genetic clustering analysis. *K* is the number of genetic clusters assumed. (a) The delta *K* plot shows multiple peaks. (b) STRUCTURE bar plot at *K* = 2, 3, 5, and 7. Each bar represents an individual *P*. *noxius* isolate. The colors represent different genetic clusters, and the lengths of the colored segments in a bar represent the estimated membership proportions of that individual to each cluster.

The result of the Bayesian clustering analysis for the overall population is in Fig 2. In the Δ*K* plot, peaks were observed at *K* = 2, 3, 5, and 7. With STRUCTURE constrained to estimate 2, 3, 5, or 7 clusters, most individuals appeared highly admixed, which suggested no significant clustering structure, at least for *K* from 1 to 20. The whole population was then arbitrarily divided into six geographical subpopulations for subsequent analyses. The global *F*_ST_ value across the six geographical subpopulations was low (*F*_ST_ = 0.015) but significantly differed from zero (*P* < 0.001) (Table 3). All pairwise *F*_ST_ values between the six subpopulations were also low (*F*_ST_ -0.0012 to 0.0126, *P* = 0.0033; S3 Table). The small overall and pairwise *F*_ST_ values supported low population differentiation in Taiwan. AMOVA revealed that 98.95% of the genetic variation observed was accounted for by the within-subpopulations rather than the among-subpopulations effects (S4 Table).

**Fig 3:**
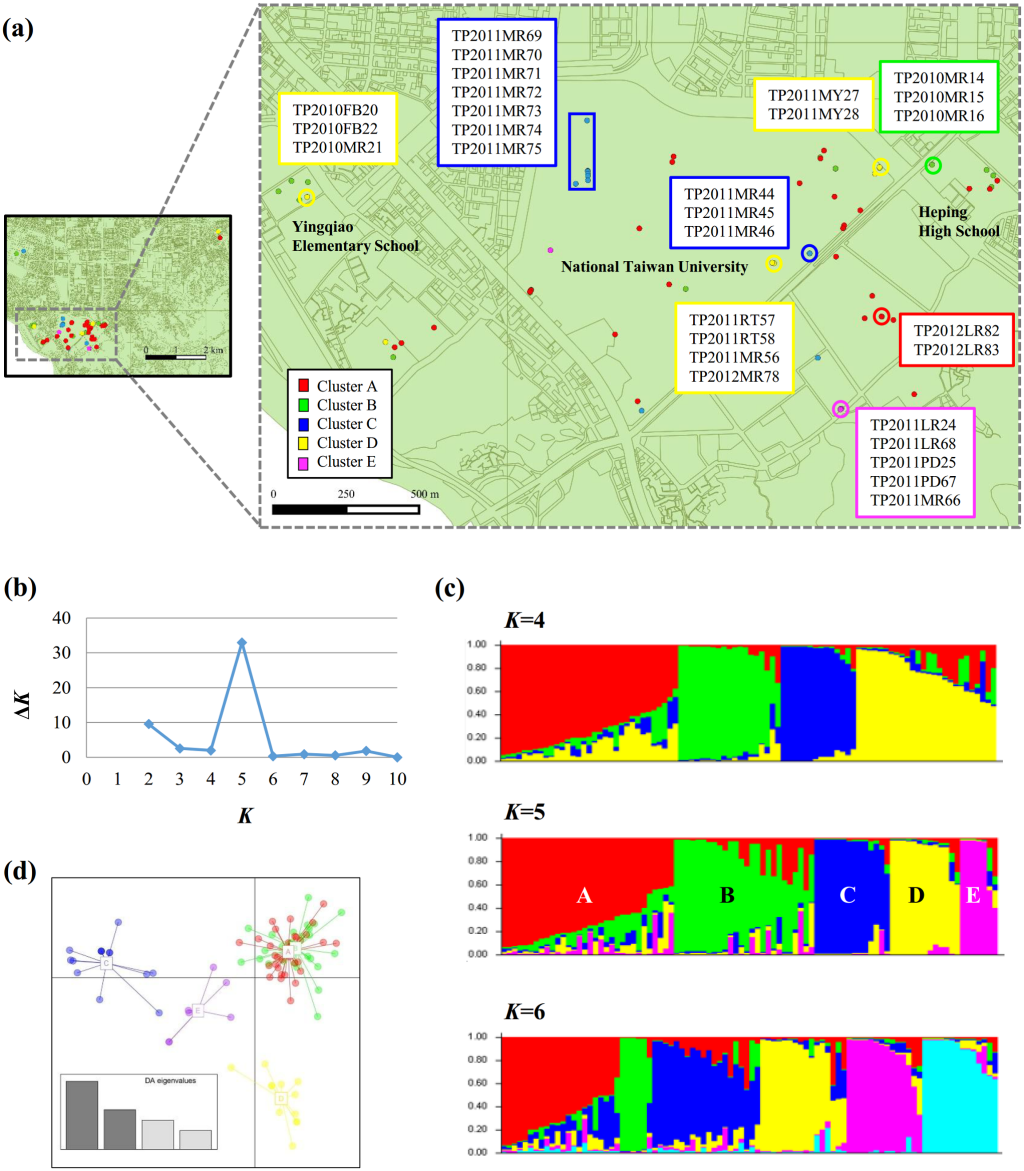
Estimated *Phellinus noxius* population structure in Taipei by Bayesian genetic clustering analysis. (a) Map of the collection sites in Taipei (left: the 7 x 4 km^2^ area containing 92 isolates; right: the 3 x 1.2 km^2^ area containing 85 isolates). Dots in different colors represent *P*. *noxius* isolates of different genetic clusters. The isolates grouped in boxes are genetically highly similar. (b) The delta *K* plot shows a clear peak at the optimal value of *K* = 5. *K* is the number of genetic clusters assumed. (c) STRUCTURE bar plot at *K* = 4, 5, and 6. Each bar represents an individual *P*. *noxius* isolate. The colors represent different genetic clusters, and the lengths of the colored segments in a bar represent the estimated membership proportions of that individual to each cluster. (d) Discriminant analysis of principal components (DAPC) for the five presumed clusters inferred by STRUCTURE analysis. The scatterplot shows only the first two principle components (PCs) accounting for 80% of the total variance. DAPC eigenvalues are illustrated in the enclosed barplot.

We performed Bayesian clustering analysis for the Taipei subpopulation. The 92 *P*. *noxius* isolates in this subpopulation were sampled from an area of 7 x 4 km^2^. Among them, 85 isolates were sampled from a small urban area of 3 x 1.2 km^2^, where three campuses are located (Fig 3a). The result in Fig 3b shows that *K* = 5 has a clear peak and thus is the most supported clustering value. In each of the five discernible genetic clusters (Fig 3c), some samples showed very high membership proportions to the cluster; the rest of the samples seemed to be genetically admixed. DAPC and *F*_ST_ were used to assess the relationship of the five presumed genetic clusters in the Taipei subpopulation. Based on DAPC (Fig 3d), clusters A and B were not separated (pairwise *F*_ST_ = 0.037), whereas clusters C, D, and E were highly differentiated (*F*_ST_ = 0.329, 0.374, and 0.305 between clusters C and D, C and E, and D and E, respectively). Moderate pairwise *F*_ST_ values were obtained between clusters A and C/D/E (*F*_ST_ = 0.130–0.163) and between clusters B and C/D/E (*F*_ST_ = 0.191–0.265). The samples assigned to a specific cluster did not necessarily originate from neighboring areas (Fig 3a). In each genetic cluster, only the isolates sharing highly similar genotypes (those showing very high membership proportions to the cluster in Fig 3c) were from diseased trees/stumps located near each other. The locations where these groups of isolates were collected can be considered different disease foci. AMOVA revealed that within-clusters rather than among-clusters effects accounted for 82.38% of the genetic variation (S4 Table).

### Spatial genetic structure

We performed analysis of isolation by distance to investigate the relationship between spatial distance and genetic similarity for the entire Taiwan population and for the six geographical subpopulations, and none of the datasets were found significant. Slopes of the regression lines were 0.005 (*P* = 0) for all Taiwan, 0.013 (*P* = 0.153) for TP, 0.018 (*P* = 0.047) for HM, 0.016 (*P* = 0.103) for CW, 0.040 (*P* = 0) for SW, 0.037 (*P* = 0) for YL, and -0.002 (*P* = 0.157) for EV. Similar non-significant patterns were observed when considering only one isolate representative of the genetically highly identical isolates collected from diseased trees/stumps located near each other.

### Phylogenetic relationships among the *P*. *noxius* isolates from different geographical areas, host plant families, and collection years

An NJ tree was constructed to illustrate the phylogenetic relationships among the 329 *P*. *noxius* isolates (S2 Fig). Low bootstrap values were detected at most of the node points, and no clustering of isolates from the same geographical areas was observed, which indicates lack of correlation between genotype and geographical locality in our dataset. Similarly, isolates from the same host plant families or collection years/time-periods were not clustered or genetically related. These findings agreed with the results of AMOVA (S4 Table) that > 96% of the genetic variation could be explained by the within-geographical-subpopulations, within-host-families or within-collection-years/time-periods components. To know the genetic variation of isolates collected from diseased trees/stumps located near each other, we examined the SSR genotypes of 60 isolates originated from 23 sites (2–7 isolates per collection site) in detail. For 5 out of 23 sites, isolates from neighboring trees/stumps at the same sites have identical genotypes. For the remaining 18 sites, isolates from neighboring trees/stumps shared high genetic similarity, different only in one of the two alleles at 1–5 SSR loci. In line with these results, isolates collected from neighboring trees/stumps are grouped in the same cluster as shown in the dendrogram (S2 Fig; marked as black asterisks).

## Discussion

The *de novo* development of SSR markers used to be laborious, time-consuming and expensive. It involved the construction of a genomic library (enriched for repeated motifs or not), isolation and sequencing of SSR containing clones, and primer design and optimization. Alternatively, SSR markers can be derived from cloning and sequencing of the amplicons of PCR-based molecular markers [44]. With the development of NGS technology and bioinformatics, obtaining a draft genome of an organism is becoming easier for genome-wide search of repeat regions and rapid SSR marker development. In recent years, this approach has allowed for efficient development of large numbers of SSR markers for a wide range of organisms, including several plant fungal and oomycete pathogens such as *Phytophthora ramorum* [45] and *Anisogramma anomala* [46]. In this study, as a first step to decipher the biology of *P*. *noxius*, we performed Illumina sequencing and *de novo* assembly of the genome of a single basidiospore isolate. Similar to the microsatellite patterns found in many other fungi (including Basidiomycetes), microsatellites identified in *P*. *noxius* genome are enriched in the short repeat motifs, and the numbers of repeat units are greater for the short than long repeat motifs [47–49]. The 13 newly developed SSR markers are locus-specific, easy to be amplified, and contain decent levels of polymorphism, for a robust and applicable codominant marker system for population genetics studies in *P*. *noxius*.

Abundant *P*. *noxius* isolates sampled across sufficiently large geographic areas and diverse host trees were used to infer the occurrence and dissemination pattern of brown root rot in Taiwan. High levels of genetic diversity were detected in the overall population and the six geographical subpopulations of *P*. *noxius* in Taiwan. There seems little to no barrier to gene flow throughout the *P*. *noxius* population in Taiwan, which may be attributed to a combined effect of basidiospore dispersal and the migration of *P*. *noxius* via infected seedlings, trees, and debris. The widespread distribution of diverse MLGs indicated that the epidemic is caused by diverse clones rather than a single predominant highly virulent strain of *P*. *noxius*. In closely related *Phellinus* species such as *P*. *linteus*, *P*. *weirii*, *P*. *gilvus*, and *P*. *tremulae*, sexual reproduction is governed by bipolar or tetrapolar heterothallic systems [50–53]. The heterothallic mating system, found in most Basidiomycota, prevents mating between identical haploids and promotes gene recombination and the generation of diversified offspring. Our molecular data revealed that all the samples were heterozygous for at least one of the 13 SSR loci, which indicated that none of the 329 isolates were genome-wide homozygous. These findings, together with the identification of a high proportion of unique MLGs, support the possibility of a predominant outcrossing reproductive mode in *P*. *noxius*. However, with the lack of clamp connections for diagnosing compatibility, the mating system of *P*. *noxius* remains to be clarified.

Our intensive sampling at three nearby campuses in Taipei further revealed that even *P*. *noxius* isolates collected within a small area of 3 x 1.2 km^2^ showed a high level of genetic diversity. Nonetheless, isolates from neighboring infected trees showed identical or nearly identical genotypes, so *P*. *noxius* might have spread from diseased to adjacent healthy trees through root-to-root contact. In support of our result, Hattori et al. (1996) identified up to 25 different *P*. *noxius* clones, mostly broadly distributed in five small sampling plots (each 15–25 × 10–20 m^2^) in a windbreak [13]. Because arthospores were hardly seen in infected tissues in the field [7], the mycelia rather than the asexual spores likely play a key role in the spread of *P*. *noxius* between neighboring trees. The reason that genotypes of some isolates from neighboring trees are not perfectly identical may attribute to the inclusion of different sets of nuclei during the process of single-hyphal-tip or single-arthospore isolation.

Analyses based on Bayesian clustering, *F*_ST_ statistics, and AMOVA all suggested low population differentiation for *P*. *noxius* in Taiwan. For each maker locus, most alleles (except for the rare ones) could be detected in different geographical subpopulations. The phylogenetic tree also showed that the isolates sampled from diverse areas of Taiwan were randomly clustered. Moreover, analysis of the isolation by distance revealed no spatial pattern of genetic diversity in all of Taiwan or within the six geographical subpopulations. Heterozygosity deficit within the population, significant deviation from Hardy-Weinberg expectation across all loci, and the high *F*_IS_ values detected in the overall population and subpopulations may reflect the fact of non-random mating, which, in our populations, may result from sibling mating, vegetative hyphal fusion, and/or sampling bias. Because we observed low population differentiation across Taiwan, and the *P*. *noxius* individuals in different areas were somewhat genetically related, the effect of inbreeding between closely related individuals (siblings) is not surprising. Vegetative hyphal fusion may be a common phenomenon and a possible source of new genetic variability in *P*. *noxius* because we could detect multinucleate and heterokaryotic individuals by SSR genotyping and DAPI staining. Small sample sizes from distinct genetic clones within the population also may have contributed to high *F*_IS_ values [54], as sampling bias could have occurred because trees infected with *P*. *noxius* can be asymptomatic, but the isolates we collected were solely from symptomatic trees or decaying stumps.

Previous studies based on (indirect) population genetic analyses or (direct) trapping of airborne spores support the involvement of basidiospores in the dissemination of many wood-decay Basidiomycetes fungi, including *Armillaria cepistipes* [55], *Armillaria mellea* [56], *Cylindrobasidium argenteum* [57], *Fomitopsis pinicola* [58], and *Serpula lacrymans* [59]. Our findings of diverse MLGs, low geographical differentiation, and lack of a clear pattern of isolation by distance also suggested a potentially important role of basidiospore dispersal in the spread of brown root rot and colonization of new habitats by *P*. *noxius*. Basidiocarps and basidiospores of *P*. *noxius* are rarely seen in the field [13]. However, in recent years, we found several cases of basidiocarps that were formed on naturally infected trees in Taiwan. Moreover, some of the basidiocarps released abundant basidiospores on rainy days (unpublished). As demonstrated by artificial inoculation, basidiospores of *P*. *noxius* were able to infect fresh stumps of hoop pine, although the rate of successful inoculation was low [7]. The distribution of basidiospores can be affected by biological characteristics (e.g., size and shape of the spore) [60] and environmental factors (e.g., temperature, humidity, wind velocity, and number of hours of bright sunshine)[61]; although most basidiospores are deposited within a limited distance (< a few meters) from the inoculum source [56,62,63], low amounts of basidiospores are thought to spread over long distances (> several kilometers) by wind. With precultivated monokaryotic colonies as baits, studies of several fungi revealed the spores of *Fomitopsis rosea*, *Cystostereum murraii* and *Phlebia centrifuga* at sites 3 km away from the nearest fruit bodies [64], the spores of *Heterobasidion annosum* trapped 50–500 km apart from the inoculum source [61], and the spores of *Peniophora aurantiaca* captured ~1000 km from a known natural occurrence [65]. Further examining the dispersal ability of *P*. *noxius* basidiospores by using available spore trapping techniques [64] would be of interest.

A disease cycle similar to other root-rotting basidiomycetes, such as *A*. *mellea*, *Ganoderma australe*, and *H*. *annosum*, was proposed for *P*. *noxius*, but the role of basidiospores has long been equivocal [1]. In agreement with the hypotheses of Hattori et al. [13], our results show that *P*. *noxius* may spread over short distances by hyphal extension via root-to-root contact of the hosts, and the genetically variable basidiospores are likely responsible for long-distance dispersal and the establishment of unique clones in new infection sites. Basidiospores may infect plants directly through fresh wounds [7] or indirectly by colonizing plant debris and extending to infect the roots of a surrounding plant. Basidiospores of *P*. *weirii*, the causal agent of laminated root rot of certain conifers, could germinate and colonize previously frozen and scalded wood disks [66]. Chang (1996) reported that *P*. *noxius* basidiospores can survive for 3 to 4.5 months in soils of varying moisture levels, and *P*. *noxius* can survive on woody debris for more than 10 years [8]. Future studies of the colonization and survival of *P*. *noxius* basidiospores on woody materials would help explain how the basidiospores can still be a key player when the occurrence of *P*. *noxius* basidiocarps is relatively low. For long-distance spread of brown root rot disease, the movement of diseased plants or debris by humans cannot be ruled out. However, the effect of human activities is difficult to evaluate because the records for cultivation and transplanting are poorly maintained in most cases. Nevertheless, the inoculum of *P*. *noxius*, originating from distant infection sites, may be more common in the environment than we imagined. Moreover, whether a tree will become diseased seems to depend largely on its health status. In urban areas in Taiwan, brown root rot disease has often been observed on weakened trees surrounded by cement or grown in small tree wells. Damage caused by transplantation and the increasing frequency of extreme weather events may also threaten the health of trees and make trees more susceptible to *P*. *noxius* infection. Overall, molecular evidence from this study provides important insight into the population genetic structure of *P*. *noxius* as well as local, regional, and distant spread of brown root rot disease caused by this fungus.

## Supporting Information

S1 Fig DAPI staining to reveal the number of nuclei present in different life stages of *Phellinus noxius*. DAPI-stained (a) mycelium, (b) arthospores, (c) basidiospores, and (d) a germinating basidiospore examined in the light- (left) and fluorescent-field (right) under microscope. (Scale bars, 10 μm).
(PPTX)

S2 Fig Phylogenic relationship of the 329 *Phellinus noxius* isolates from 14 cities/counties in Taiwan from 1989 to 2012. The neighbor-joining (NJ) tree was constructed on the basis of the genotypes of 13 SSR markers. Numbers at branch points refer to bootstrap values (2000 iterations). Isolate IDs in different colors indicate that they were from different geographical areas. The black asterisks indicate the 60 *P*. *noxius* samples isolated from neighboring trees/stumps at 23 sites.
(PPTX)

S1 Table The 329 isolates of *Phellinus noxius* used in this study. (DOCX)

S2 Table The SSR genotypes of the 329 *Phellinus noxius* isolates. The allelic fragments for SSR markers are shown as the sizes (bp) of PCR products. For each SSR locus, only the alleles with the first and second greatest fluorescence intensity (data in the first two columns) were used for analyses in the study. The symbol "?" denotes missing data.
(XLSX)

S3 Table Pairwise *F*_ST_ values between the six geographical subpopulations. TP: Taipei; HM: Hsinchu-Miaoli Hills, CW: Central West, SW: Southern West, YL: Yilan, and WV: East Rift Valley.
(DOCX)

S4 Table Results of the analysis of molecular variance (AMOVA) assessing the proportions of genetic variations explained by the effects of geographical subpopulations, families of host trees, and collection years/time periods. (DOCX)
