# Supplementary material for: The Genetic Structure of Phellinus noxius and Dissemination Pattern of Brown Root Rot Disease in Taiwan
Source: PLoS One. 2015 Oct 20;10(10):e0139445. doi: 10.1371/journal.pone.0139445 (PMC4615629; doi:10.1371/journal.pone.0139445)
Supplement: S1 Fig — DAPI-stained (a) mycelium, (b) arthospores, (c) basidiospores, and (d) a germinating basidiospore examined in the light- (left) and fluorescent-field (right) under microscope. (Scale bars, 10 μm). (PPTX) [file pone.0139445.s001.pptx]

## Slide 1
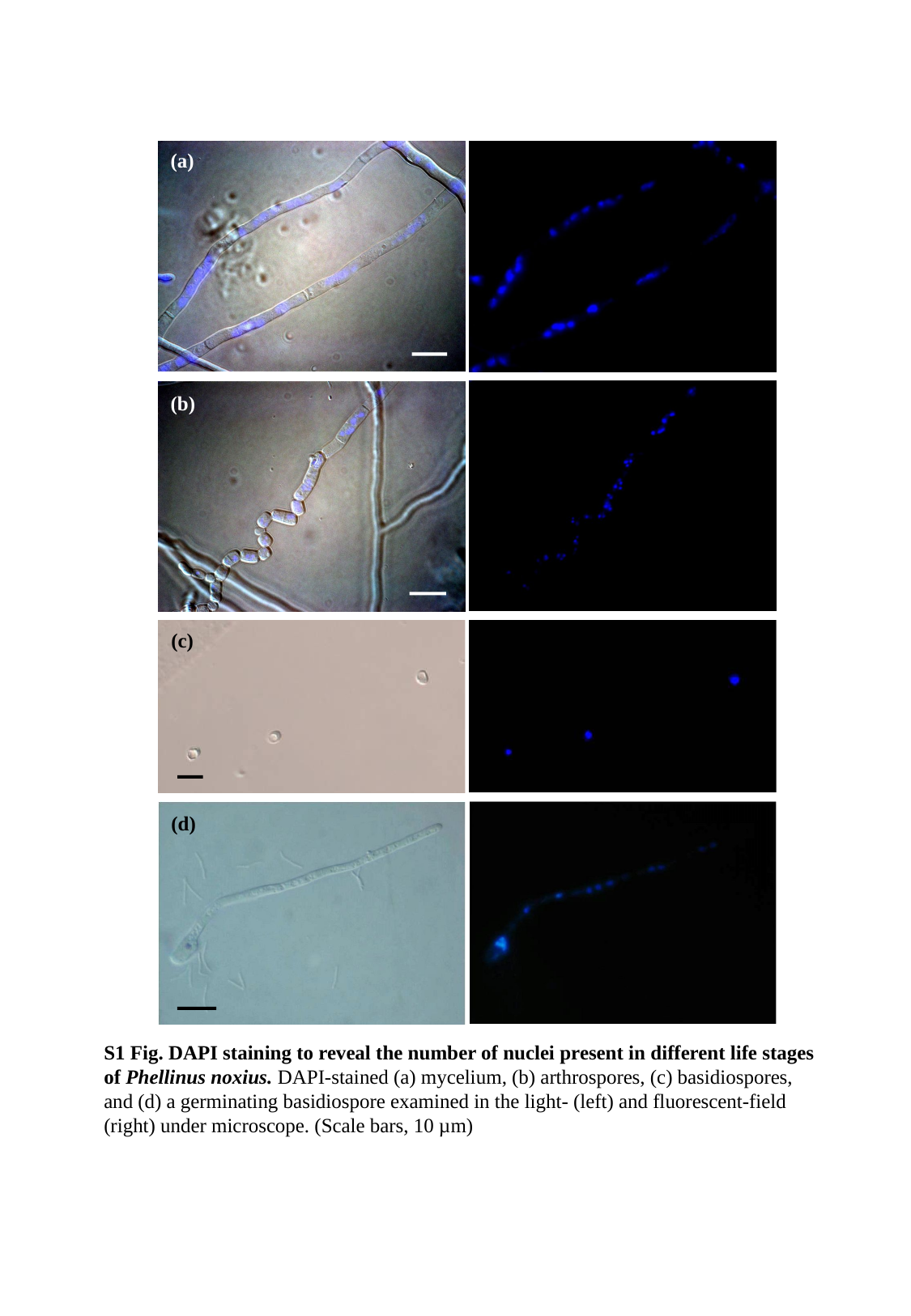

(a)
(b)
(c)
(d)
S1 Fig. DAPI staining to reveal the number of nuclei present in different life stages of Phellinus noxius. DAPI-stained (a) mycelium, (b) arthrospores, (c) basidiospores, and (d) a germinating basidiospore examined in the light- (left) and fluorescent-field (right) under microscope. (Scale bars, 10 µm)
