# Supplementary material for: The Genetic Structure of Phellinus noxius and Dissemination Pattern of Brown Root Rot Disease in Taiwan
Source: PLoS One. 2015 Oct 20;10(10):e0139445. doi: 10.1371/journal.pone.0139445 (PMC4615629; doi:10.1371/journal.pone.0139445)
Supplement: S2 Fig — The neighbor-joining (NJ) tree was constructed on the basis of the genotypes of 13 SSR markers. Numbers at branch points refer to bootstrap values (2000 iterations). Isolate IDs in different colors indicate that they were from different geographical areas. The black asterisks indicate the 60 P. noxius samples isolated from neighboring trees/stumps at 23 sites. (PPTX) [file pone.0139445.s002.pptx]

## Slide 1
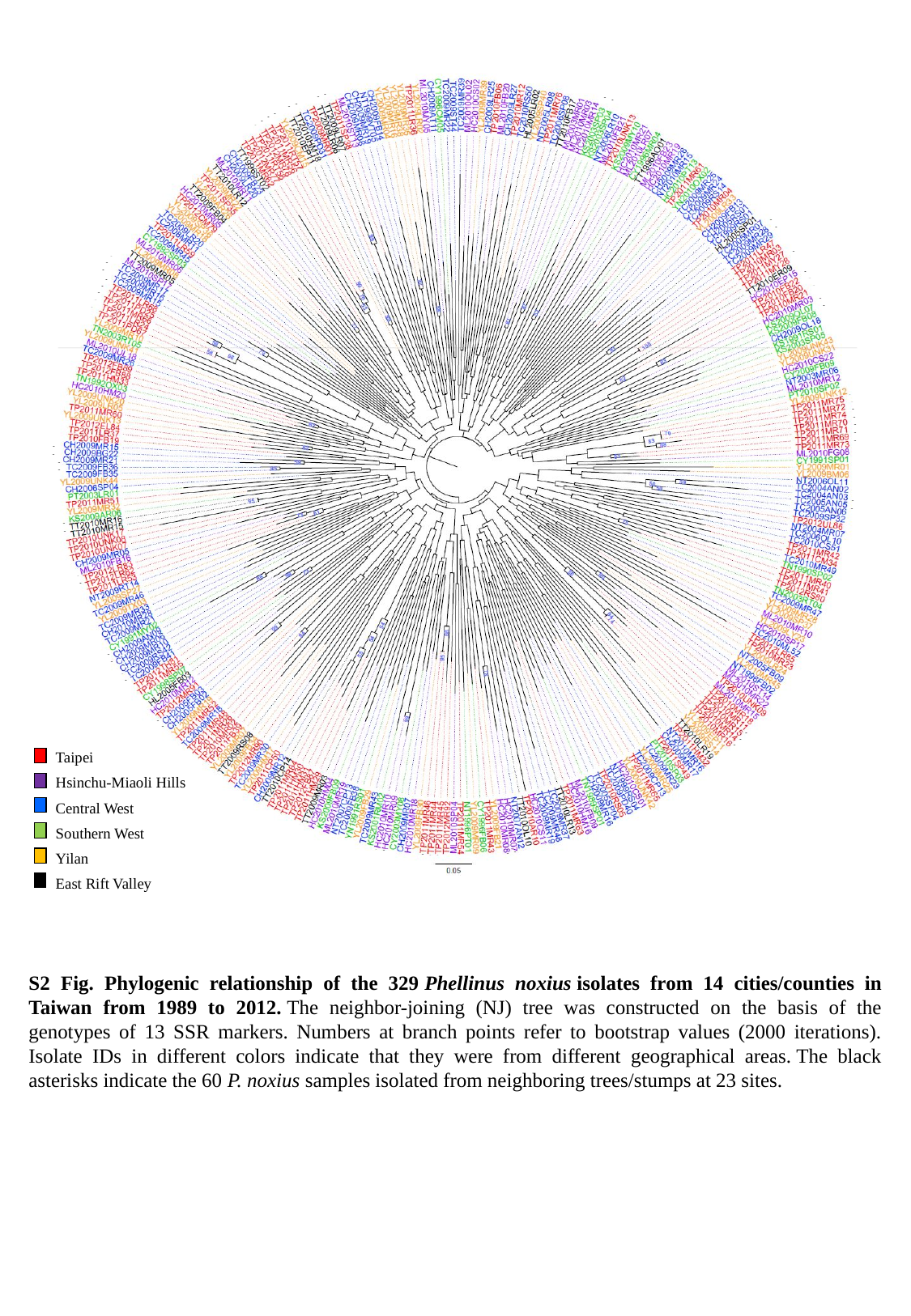

Taipei
Hsinchu-Miaoli Hills
Central West
Southern West
Yilan
East Rift Valley
S2 Fig. Phylogenic relationship of the 329 Phellinus noxius isolates from 14 cities/counties in Taiwan from 1989 to 2012. The neighbor-joining (NJ) tree was constructed on the basis of the genotypes of 13 SSR markers. Numbers at branch points refer to bootstrap values (2000 iterations). Isolate IDs in different colors indicate that they were from different geographical areas. The black asterisks indicate the 60 P. noxius samples isolated from neighboring trees/stumps at 23 sites.
