# Supplementary material for: The Genetic Structure of Phellinus noxius and Dissemination Pattern of Brown Root Rot Disease in Taiwan
Source: PLoS One. 2015 Oct 20;10(10):e0139445. doi: 10.1371/journal.pone.0139445 (PMC4615629; doi:10.1371/journal.pone.0139445)
Supplement: S1 Table — (DOCX) [file pone.0139445.s003.docx]

**S1 Table. The 329 isolates of *Phenillus noxius* used in this study.**

| Isolate ID ^a^ | Collection  year | City/County | Host plant ^b^ | Source ^c^ |
| --- | --- | --- | --- | --- |
| TC1999RS01 | 1999 | Taichung | *Prunus persica* | TARI |
| TC2004AN02 | 2004 | Taichung | *Schinus terebinthifolinus* | TARI |
| TC2004AN03 | 2004 | Taichung | *Schinus terebinthifolinus* | TARI |
| TC2005SP04 | 2005 | Taichung | *Dimocarpus longan* | TARI |
| TC2005AN05 | 2005 | Taichung | *Schinus terebinthifolinus* | TARI |
| TC2005AN06 | 2005 | Taichung | *Schinus terebinthifolinus* | TARI |
| TC2005MR07 | 2005 | Taichung | *Ficus* sp. | TARI |
| TC2006SP08 | 2006 | Taichung | *Dimocarpus longan* | TARI |
| TC2006MR09 | 2006 | Taichung | *Ficus* sp. | TARI |
| TC2006OL10 | 2006 | Taichung | *Osmanthus fragrans* | TARI |
| TC2008MR11 | 2008 | Taichung | *Ficus* sp. | TARI |
| TC2008ST12 | 2008 | Taichung | *Sterculia nobilis* | TARI |
| TC2009CP13 | 2009 | Taichung | *Ficus* sp. | TARI |
| TC2009RS14 | 2009 | Taichung | *Ficus* sp. | TARI |
| TC2009MR15 | 2009 | Taichung | *Ficus* sp. | TARI |
| TC2009MR16 | 2009 | Taichung | *Calocedrus formosana* | TARI |
| TC2009MR17 | 2009 | Taichung | *Prunus serrulata* | TARI |
| TC2009MR18 | 2009 | Taichung | *Ficus* sp. | TARI |
| TC2009MR19 | 2009 | Taichung | *Ficus* sp. | TARI |
| TC2009LR20 | 2009 | Taichung | *Ficus* sp. | TARI |
| TC2009MR21 | 2009 | Taichung | *Ficus* sp. | TARI |
| TC2009MR22 | 2009 | Taichung | *Ficus* sp. | TARI |
| TC2009MR23 | 2009 | Taichung | *Cinnamomum camphora* | TARI |
| TC2009MR24 | 2009 | Taichung | *Ficus benjamina* | TARI |
| TC2009MR25 | 2009 | Taichung | *Ficus* sp. | TARI |
| TC2009MR26 | 2009 | Taichung | *Ficus benjamina* | TARI |
| TC2009SP27 | 2009 | Taichung | *Ficus religiosa* | TARI |
| TC2009MR28 | 2009 | Taichung | *Ficus benjamina* | TARI |
| TC2009MR29 | 2009 | Taichung | *Ficus benjamina* | TARI |
| TC2009MR30 | 2009 | Taichung | *Koelreuteria elegans* | TARI |
| TC2009PD31 | 2009 | Taichung | *Ficus* sp. | TARI |
| TC2009SP32 | 2009 | Taichung | *Ficus* sp. | TARI |
| TC2009MR33 | 2009 | Taichung | *Ficus* sp. | TARI |
| TC2009SP34 | 2009 | Taichung | *Podocarpus macrophyllus* | TARI |
| TC2009FB35 | 2009 | Taichung | *Koelreuteria elegans* | TARI |
| TC2009FB36 | 2009 | Taichung | *Ficus benjamina* | TARI |
| TC2009MR37 | 2009 | Taichung | *Koelreuteria elegans* | TARI |
| TC2009FB38 | 2009 | Taichung | *Pterocarpus indicus* | TARI |
| TC2009MR39 | 2009 | Taichung | *Pterocarpus indicus* | TARI |
| TC2009PD40 | 2009 | Taichung | *Ficus* sp. | TARI |
| TC2009FB41 | 2009 | Taichung | *Bauhinia variegata* | TARI |
| TC2009RS42 | 2009 | Taichung | *Ficus superba* | TARI |
| TC2009MR43 | 2009 | Taichung | *Nageia nagi* | TARI |
| TC2009ST44 | 2009 | Taichung | *Bauhinia variegata* | TARI |
| TC2009MR45 | 2009 | Taichung | *Prunus serrulata* | TARI |
| TC2009MR46 | 2009 | Taichung | *Ficus* sp. | TARI |
| TC2009MR47 | 2009 | Taichung | *Sterculia nobilis* | TARI |
| TC2009MR48 | 2009 | Taichung | *Ficus* sp. | TARI |
| TC2010MR49 | 2010 | Taichung | *Pyrus* sp. | TARI |
| TC2010RS50 | 2010 | Taichung | *Casuarina equisetifolia* | TARI |
| TC2010CS51 | 2010 | Taichung | *Toona sinensis* | TARI |
| TC2010ML52 | 2010 | Taichung | *Ficus benjamina* | TARI |
| TP2009MR01 | 2009 | Taipei | *Ficus* sp. | TARI |
| TP2010MR02 | 2010 | Taipei | N/A | NTU |
| TP2010MR03 | 2010 | Taipei | N/A | NTU |
| TP2010MR04 | 2010 | Taipei | *Chrysalidocarpus lutescens* | NTU |
| TP2010RS05 | 2010 | Taipei | *Ficus* sp. | NTU |
| TP2010FB06 | 2010 | Taipei | *Ficus* sp. | NTU |
| TP2010UNK07 | 2010 | Taipei | N/A | NTU |
| TP2010UNK08 | 2010 | Taipei | *Ficus* sp. | NTU |
| TP2010UNK09 | 2010 | Taipei | *Ficus* sp. | NTU |
| TP2010AR10 | 2010 | Taipei | *Ficus* sp. | NTU |
| TP2010MR11 | 2010 | Taipei | N/A | NTU |
| TP2010MR12 | 2010 | Taipei | *Ficus microcarpa* cv. GoldenLeaves | NTU |
| TP2010UNK13 | 2010 | Taipei | *Delonix regia* | NTU |
| TP2010MR14 | 2010 | Taipei | *Bauhinia variegata* | NTU |
| TP2010MR15 | 2010 | Taipei | *Ficus microcarpa* | NTU |
| TP2010MR16 | 2010 | Taipei | *Ficus* sp. | NTU |
| TP2010UNK17 | 2010 | Taipei | *Bauhinia variegata* | NTU |
| TP2010MR18 | 2010 | Taipei | *Ficus* sp. | NTU |
| TP2010FB19 | 2010 | Taipei | *Ficus* sp. | NTU |
| TP2010FB20 | 2010 | Taipei | *Morus australis* | NTU |
| TP2010MR21 | 2010 | Taipei | *Prunus campanulata* | NTU |
| TP2010FB22 | 2010 | Taipei | *Bauhinia variegata* | NTU |
| TP2010MR23 | 2010 | Taipei | N/A | NTU |
| TP2011LR24 | 2011 | Taipei | *Terminalia boivinii* | NTU |
| TP2011PD25 | 2011 | Taipei | *Dimocarpus longan* | NTU |
| TP2011MR26 | 2011 | Taipei | *Cinnamomum kotoense* | NTU |
| TP2011MY27 | 2011 | Taipei | *Cinnamomum kotoense* | NTU |
| TP2011MY28 | 2011 | Taipei | *Koelreuteria elegans* | NTU |
| TP2011HM29 | 2011 | Taipei | *Delonix regia* | NTU |
| TP2011HM30 | 2011 | Taipei | *Ficus microcarpa* | NTU |
| TP2011HM31 | 2011 | Taipei | *Ficus microcarpa* | NTU |
| TP2011HM32 | 2011 | Taipei | *Ficus microcarpa* | NTU |
| TP2011HM33 | 2011 | Taipei | *Ficus microcarpa* | NTU |
| TP2011CM34 | 2011 | Taipei | *Ficus microcarpa* | NTU |
| TP2011SP35 | 2011 | Taipei | *Ficus microcarpa* | NTU |
| TP2011LR36 | 2011 | Taipei | *Ficus microcarpa* | NTU |
| TP2011LR37 | 2011 | Taipei | *Cinnamomum camphora* | NTU |
| TP2011SP38 | 2011 | Taipei | *Ficus microcarpa* | NTU |
| TP2011FB39 | 2011 | Taipei | *Koelreuteria elegans* | NTU |
| TP2011MR40 | 2011 | Taipei | *Juniperus chinensis* var. *kaizuka* | NTU |
| TP2011MR41 | 2011 | Taipei | *Ficus superba* | NTU |
| TP2011MR42 | 2011 | Taipei | *Ficus  elastica* | NTU |
| TP2011MR43 | 2011 | Taipei | *Cinnamomum camphora* | NTU |
| TP2011MR44 | 2011 | Taipei | *Ficus  elastica* | NTU |
| TP2011MR45 | 2011 | Taipei | *Ficus microcarpa* | NTU |
| TP2011MR46 | 2011 | Taipei | *Ficus benjamina* | NTU |
| TP2011LR47 | 2011 | Taipei | *Murraya paniculata* | NTU |
| TP2011MR48 | 2011 | Taipei | *Murraya paniculata* | NTU |
| TP2011SP49 | 2011 | Taipei | *Cinnamomum camphora* | NTU |
| TP2011CP50 | 2011 | Taipei | *Ficus microcarpa* | NTU |
| TP2011MR51 | 2011 | Taipei | *Ficus microcarpa* | NTU |
| TP2011MR52 | 2011 | Taipei | *Ficus microcarpa* | NTU |
| TP2011LR53 | 2011 | Taipei | *Ficus microcarpa* | NTU |
| TP2011MR54 | 2011 | Taipei | *Ficus superba* | NTU |
| TP2011MR55 | 2011 | Taipei | *Ficus superba* | NTU |
| TP2011MR56 | 2011 | Taipei | *Ficus elastica* | NTU |
| TP2011RT57 | 2011 | Taipei | *Nageia nagi* | NTU |
| TP2011RT58 | 2011 | Taipei | *Cinnamomum camphora* | NTU |
| TP2011LR59 | 2011 | Taipei | *Ficus benjamina* | NTU |
| TP2011MR60 | 2011 | Taipei | *Ficus benjamina* | NTU |
| TP2011MR61 | 2011 | Taipei | *Ficus benjamina* | NTU |
| TP2011MR62 | 2011 | Taipei | *Ficus benjamina* | NTU |
| TP2011MR63 | 2011 | Taipei | *Ficus benjamina* | NTU |
| TP2011MR64 | 2011 | Taipei | *Ficus benjamina* | NTU |
| TP2011MR65 | 2011 | Taipei | *Ficus benjamina* | NTU |
| TP2011MR66 | 2011 | Taipei | *Ficus microcarpa* | NTU |
| TP2011PD67 | 2011 | Taipei | *Cinnamomum camphora* | NTU |
| TP2011LR68 | 2011 | Taipei | *Nageia nagi* | NTU |
| TP2011MR69 | 2011 | Taipei | *Ficus* sp. | NTU |
| TP2011MR70 | 2011 | Taipei | *Eucalyptus robusta* | NTU |
| TP2011MR71 | 2011 | Taipei | *Eucalyptus robusta* | NTU |
| TP2011MR72 | 2011 | Taipei | *Liquidambar formosana* | NTU |
| TP2011MR73 | 2011 | Taipei | *Liquidambar formosana* | NTU |
| TP2011MR74 | 2011 | Taipei | *Liquidambar formosana* | NTU |
| TP2011MR75 | 2011 | Taipei | *Liquidambar formosana* | NTU |
| TP2011MR76 | 2011 | Taipei | *Liquidambar formosana* | NTU |
| TP2012MR77 | 2012 | Taipei | *Ficus benjamina* | NTU |
| TP2012MR78 | 2012 | Taipei | *Ficus benjamina* | NTU |
| TP2012CM79 | 2012 | Taipei | *Ficus microcarpa* | NTU |
| TP2012RS80 | 2012 | Taipei | *Ficus microcarpa* | NTU |
| TP2012TH81 | 2012 | Taipei | *Ficus benjamina* | NTU |
| TP2012LR82 | 2012 | Taipei | *Terminalia boivinii* | NTU |
| TP2012LR83 | 2012 | Taipei | *Prunus campanulata* | NTU |
| TP2012EL84 | 2012 | Taipei | *Cleyera japonica* var. *morii* | NTU |
| TP2012LR85 | 2012 | Taipei | *Cinnamomum camphora* | NTU |
| TP2012UL86 | 2012 | Taipei | *Cinnamomum camphora* | NTU |
| TP2012FB87 | 2012 | Taipei | *Elaeocarpus sylvestris* | NTU |
| TP2012FB88 | 2012 | Taipei | *Cinnamomum camphora* | NTU |
| TP2012FB89 | 2012 | Taipei | *Zelkova formosana* | NTU |
| TP2012MR90 | 2012 | Taipei | *Delonix regia* | NTU |
| TP2012MR91 | 2012 | Taipei | *Delonix regia* | NTU |
| TP2012MR92 | 2012 | Taipei | *Delonix regia* | NTU |
| TT1996AO01 | 1996 | Taitung | *Annona squamosa* | TARI |
| TT1996ST02 | 1996 | Taitung | *Sterculia nobilis* | TARI |
| TT2009MR03 | 2009 | Taitung | *Ficus* sp. | TTDARES |
| TT2009FB04 | 2009 | Taitung | *Pterocarpus indicus* | TTDARES |
| TT2009MR05 | 2009 | Taitung | *Cinnamomum camphora* | TTDARES |
| TT2009LR06 | 2009 | Taitung | *Cinnamomum kotoense* | TARI |
| TT2009LR07 | 2009 | Taitung | *Cinnamomum kotoense* | TARI |
| TT2009RS08 | 2009 | Taitung | *Eriobotrya japonica* | TARI |
| TT2010ER09 | 2010 | Taitung | *Cassia fistula* | TTDARES |
| TT2010OL10 | 2010 | Taitung | *Liquidambar formosana* | TTDARES |
| TT2010EP11 | 2010 | Taitung | *Cinnamomum kanehirae* | TTDARES |
| TT2010LR12 | 2010 | Taitung | *Ixora* x *williamsii* | TTDARES |
| TT2010LR13 | 2010 | Taitung | *Cinnamomoum osmophloeum* | TTDARES |
| TT2010CP14 | 2010 | Taitung | *Cinnamomum kanehirae* | TTDARES |
| TT2010MR15 | 2010 | Taitung | *Juniperus chinensis* var. *kaizuka* | TTDARES |
| TT2010MR16 | 2010 | Taitung | *Ficus pumlia* | TTDARES |
| TT2010FB17 | 2010 | Taitung | *Ficus pumlia* | TTDARES |
| TT2010HM18 | 2010 | Taitung | *Rhododendron  simsii* | TTDARES |
| TT2010LR19 | 2010 | Taitung | *Osmanthus fragrans* | TTDARES |
| TN1989SP01 | 1989 | Tainan | *Dimocarpus longan* | TARI |
| TN1990SP02 | 1990 | Tainan | *Dimocarpus longan* | TARI |
| TN1992OX03 | 1992 | Tainan | *Averrhoa carambola* | TARI |
| TN2003RT04 | 2003 | Tainan | *Citrus limonia* | TARI |
| TN2003RT05 | 2003 | Tainan | *Citrus limonia* | TARI |
| YL2009MR01 | 2009 | Yilan | *Ficus* sp. | TFRI |
| YL2009LR02 | 2009 | Yilan | *Cinnamomum camphora* | TFRI |
| YL2009TX03 | 2009 | Yilan | *Taxodium distichum* | TFRI |
| YL2009MR04 | 2009 | Yilan | *Ficus* sp. | TFRI |
| YL2009LR05 | 2009 | Yilan | *Cinnamomum camphora* | TFRI |
| YL2009BM06 | 2009 | Yilan | *Ficus* sp. | TFRI |
| YL2009MR07 | 2009 | Yilan | *Ficus* sp. | TFRI |
| YL2009MR08 | 2009 | Yilan | *Ficus* sp. | TFRI |
| YL2009MR09 | 2009 | Yilan | *Ficus religiosa* | TFRI |
| YL2009MR10 | 2009 | Yilan | *Ficus* sp. | TFRI |
| YL2009EP11 | 2009 | Yilan | *Bischofia jabanica* | TFRI |
| YL2009UNK12 | 2009 | Yilan | N/A | TFRI |
| YL2009UNK13 | 2009 | Yilan | N/A | TFRI |
| YL2009RS14 | 2009 | Yilan | *Prunus campanulata* | TFRI |
| YL2009CM15 | 2009 | Yilan | *Terminalia boivinii* | TFRI |
| YL2009MR16 | 2009 | Yilan | *Ficus microcarpa* cv.GoldenLeaves | TFRI |
| YL2009HM17 | 2009 | Yilan | *Liquidambar formosana* | TFRI |
| YL2009MR18 | 2009 | Yilan | *Ficus* sp. | TFRI |
| YL2009MR19 | 2009 | Yilan | *Ficus religiosa* | TFRI |
| YL2009UNK20 | 2009 | Yilan | N/A | TFRI |
| YL2009FB21 | 2009 | Yilan | N/A | TFRI |
| YL2009MR22 | 2009 | Yilan | *Bauhinia variegata* | TFRI |
| YL2009LY23 | 2009 | Yilan | *Ficus* sp. | TFRI |
| YL2009LR24 | 2009 | Yilan | *Lagerstroemia micrantha* | TFRI |
| YL2009MR25 | 2009 | Yilan | *Cinnamomum camphora* | TFRI |
| YL2009AR26 | 2009 | Yilan | *Ficus* sp. | TFRI |
| YL2009SP27 | 2009 | Yilan | *Roystonea regia* | TFRI |
| YL2009MR28 | 2009 | Yilan | *Koelreuteria elegans* | TFRI |
| YL2009FB29 | 2009 | Yilan | *Ficus* sp. | TFRI |
| YL2009FB30 | 2009 | Yilan | *Cassia fistula* | TFRI |
| YL2009UNK31 | 2009 | Yilan | *Bauhinia variegata* | TFRI |
| YL2009MR32 | 2009 | Yilan | N/A | TFRI |
| YL2009LR33 | 2009 | Yilan | *Ficus* sp. | TFRI |
| YL2009FB34 | 2009 | Yilan | *Cinnamomum  verum* | TFRI |
| YL2009MR35 | 2009 | Yilan | *Delonix regia* | TFRI |
| YL2009SP36 | 2009 | Yilan | *Ficus* sp. | TFRI |
| YL2009SP37 | 2009 | Yilan | *Koelreuteria elegans* | TFRI |
| YL2009MR38 | 2009 | Yilan | *Koelreuteria elegans* | TFRI |
| YL2009MR39 | 2009 | Yilan | *Ficus* sp. | TFRI |
| YL2009SP40 | 2009 | Yilan | *Ficus* sp. | TFRI |
| YL2009UNK41 | 2009 | Yilan | *Koelreuteria elegans* | TFRI |
| YL2009UNK42 | 2009 | Yilan | N/A | TFRI |
| YL2009UNK43 | 2009 | Yilan | N/A | TFRI |
| YL2009UNK44 | 2009 | Yilan | N/A | TFRI |
| YL2010MR45 | 2010 | Yilan | *Ficus* sp. | TFRI |
| HL2005SP01 | 2005 | Hualien | *Koelreuteria elegans* | TARI |
| HL2005LR02 | 2005 | Hualien | *Cinnamomum camphora* | TARI |
| HL2005FB03 | 2005 | Hualien | *Bauhinia variegata* | TARI |
| NT1996PT01 | 1996 | Nantou | *Grevillea robusta* | TARI |
| NT1996FB02 | 1996 | Nantou | *Bauhinia variegata* | TARI |
| NT1996RS03 | 1996 | Nantou | *Prunus mume* | TARI |
| NT1999RS04 | 1999 | Nantou | *Prunus persica* | TARI |
| NT1999VT05 | 1999 | Nantou | *Vitis* sp. | TARI |
| NT2003MR06 | 2003 | Nantou | *Ficus* sp. | TARI |
| NT2004MR07 | 2004 | Nantou | *Ficus* sp. | TARI |
| NT2005LR08 | 2005 | Nantou | *Delonix regia* | TARI |
| NT2005FB09 | 2005 | Nantou | *Cinnamomum camphora* | TARI |
| NT2006LR10 | 2006 | Nantou | *Osmanthus fragrans* | TARI |
| NT2006OL11 | 2006 | Nantou | *Cinnamomum cassia* | TARI |
| NT2007AN12 | 2007 | Nantou | *Mangifera indica* | TARI |
| NT2007CP13 | 2007 | Nantou | *Juniperus chinensis* var. *kaizuka* | TARI |
| NT2009RT14 | 2009 | Nantou | *Murraya paniculata* | TARI |
| NT2010MR15 | 2010 | Nantou | *Cinnamomum camphora* | TARI |
| NT2010MR16 | 2010 | Nantou | *Ficus benjamina* | TARI |
| PT2003LR01 | 2003 | Pintung | *Persea americana* | TARI |
| PT2010SP02 | 2010 | Pintung | *Litchi chinensis* | TARI |
| PT2010SP03 | 2010 | Pintung | *Litchi chinensis* | TARI |
| ML2003MR01 | 2003 | Miaoli | *Ficus* sp. | TARI |
| ML2010OL02 | 2010 | Miaoli | *Ficus* sp. | TARI |
| ML2010MR03 | 2010 | Miaoli | *Dimocarpus longan* | TARI |
| ML2010SP04 | 2010 | Miaoli | *Eucalyptus robusta* | TARI |
| ML2010MY05 | 2010 | Miaoli | *Ficus* sp. | TARI |
| ML2010MR06 | 2010 | Miaoli | *Cinnamomum camphora* | TARI |
| ML2010LR07 | 2010 | Miaoli | *Quercus* sp. | TARI |
| ML2010FG08 | 2010 | Miaoli | *Bauhinia variegata* | TARI |
| ML2010FB09 | 2010 | Miaoli | *Ficus* sp. | TARI |
| ML2010MR10 | 2010 | Miaoli | *Ficus benjamina* | TARI |
| ML2010MR11 | 2010 | Miaoli | *Ficus* sp. | TARI |
| ML2010MR12 | 2010 | Miaoli | *Sterculia foetida* | TARI |
| ML2010ST13 | 2010 | Miaoli | *Sterculia foetida* | TARI |
| ML2010ST14 | 2010 | Miaoli | *Ficus* sp. | TARI |
| ML2010MR15 | 2010 | Miaoli | *Bauhinia* × *blakeana* | TARI |
| ML2010FB16 | 2010 | Miaoli | *Koelreuteria elegans* | TARI |
| ML2010SP17 | 2010 | Miaoli | *Zelkova serrata* | TARI |
| ML2010UL18 | 2010 | Miaoli | *Ficus benjamina* | TARI |
| ML2010MR19 | 2010 | Miaoli | *Bauhinia variegata* | TARI |
| ML2010FB20 | 2010 | Miaoli | *Bauhinia variegata* | TARI |
| ML2010FB21 | 2010 | Miaoli | *Ficus* sp. | TARI |
| ML2010MR22 | 2010 | Miaoli | *Delonix regia* | TARI |
| ML2010FB23 | 2010 | Miaoli | *Osmanthus fragrans* | TARI |
| KS1991RS01 | 1991 | Kaohsiung | *Prunus mume* | TARI |
| KS2001RM02 | 2001 | Kaohsiung | *Zizyphus mauritiana* | TARI |
| KS2003SP03 | 2003 | Kaohsiung | *Litchi chinensis* | TARI |
| KS2003SP04 | 2003 | Kaohsiung | *Litchi chinensis* | TARI |
| KS2003SP05 | 2003 | Kaohsiung | *Litchi chinensis* | TARI |
| KS2009AR06 | 2009 | Kaohsiung | *Psidium guajava* | TARI |
| KS2009OL07 | 2009 | Kaohsiung | *Fraxinus formosana* | TFRI |
| KS2009FB08 | 2009 | Kaohsiung | *Bauhinia variegata* | TFRI |
| KS2009FB09 | 2009 | Kaohsiung | *Ixora chinensis* | TFRI |
| KS2009MY10 | 2009 | Kaohsiung | *Chrysalidocarpus lutescens* | TFRI |
| YN1991RS01 | 1991 | Yuanlin | *Eriobotrya japonica* | TARI |
| YN2010OX02 | 2010 | Yuanlin | *Averrhoa carambola* | TARI |
| HC2010CS01 | 2010 | Hsinchu | *Casuarina equisetifolia* | TARI |
| HC2010CS02 | 2010 | Hsinchu | *Casuarina equisetifolia* | TARI |
| HC2010MR03 | 2010 | Hsinchu | *Ficus* sp. | TARI |
| HC2010HM04 | 2010 | Hsinchu | *Liquidambar formosana* | TARI |
| HC2010MR05 | 2010 | Hsinchu | *Ficus* sp. | TARI |
| HC2010MR06 | 2010 | Hsinchu | *Ficus* sp. | TARI |
| HC2010MR07 | 2010 | Hsinchu | *Ficus* sp. | TARI |
| HC2010MR08 | 2010 | Hsinchu | *Ficus* sp. | TARI |
| HC2010MR09 | 2010 | Hsinchu | *Ficus* sp. | TARI |
| HC2010MR10 | 2010 | Hsinchu | *Ficus* sp. | TARI |
| HC2010CS11 | 2010 | Hsinchu | *Casuarina equisetifolia* | TARI |
| HC2010MR12 | 2010 | Hsinchu | *Ficus* sp. | TARI |
| HC2010PT13 | 2010 | Hsinchu | *Grevillea robusta* | TARI |
| HC2010MR14 | 2010 | Hsinchu | *Ficus* sp. | TARI |
| HC2010EP15 | 2010 | Hsinchu | *Bischofia jabanica* | TARI |
| HC2010HM16 | 2010 | Hsinchu | *Liquidambar formosana* | TARI |
| HC2010SP17 | 2010 | Hsinchu | *Koelreuteria elegans* | TARI |
| HC2010MR18 | 2010 | Hsinchu | *Ficus* sp. | TARI |
| HC2010MR19 | 2010 | Hsinchu | *Ficus* sp. | TARI |
| HC2010HM20 | 2010 | Hsinchu | *Liquidambar formosana* | TARI |
| HC2010MR21 | 2010 | Hsinchu | *Ficus* sp. | TARI |
| HC2010CS22 | 2010 | Hsinchu | *Casuarina equisetifolia* | TARI |
| CY1991SP01 | 1991 | Chiayi | *Dimocarpus longan* | TARI |
| CY1991MY02 | 1991 | Chiayi | *Syzygium samarangense* | TARI |
| CY1992SP03 | 1992 | Chiayi | *Litchi chinensis* | TARI |
| CY1996MR04 | 1996 | Chiayi | *Cassia fistula* | TARI |
| CY1996CM05 | 1996 | Chiayi | *Ficus pumila* var. *awkeotsang* | TARI |
| CY1996FB06 | 1996 | Chiayi | *Terminalia boivinii* | TARI |
| CY1998SP07 | 1998 | Chiayi | *Litchi chinensis* | TARI |
| CY2003MY08 | 2003 | Chiayi | *Syzygium samarangense* | TARI |
| CY2009FB09 | 2009 | Chiayi | *Delonix regia* | TARI |
| CH2005RS01 | 2005 | Changhua | *Prunus persica* | TARI |
| CH2005FB02 | 2005 | Changhua | *Bauhinia* x *blakeana* | TARI |
| CH2005FB03 | 2005 | Changhua | *Bauhinia* x *blakeana* | TARI |
| CH2006SP04 | 2006 | Changhua | *Dimocarpus longan* | TARI |
| CH2009MR05 | 2009 | Changhua | *Cinnamomum camphora* | TARI |
| CH2009MR06 | 2009 | Changhua | *Ficus* sp. | TARI |
| CH2009MR07 | 2009 | Changhua | *Ficus* sp. | TARI |
| CH2009MR08 | 2009 | Changhua | *Ficus* sp. | TARI |
| CH2009AN09 | 2009 | Changhua | *Ficus* sp. | TARI |
| CH2009MR10 | 2009 | Changhua | *Mangifera indica* | TARI |
| CH2009AT11 | 2009 | Changhua | *Ficus* sp. | TARI |
| CH2009MR12 | 2009 | Changhua | *Araucaria cunninghamii* | TARI |
| CH2009FB13 | 2009 | Changhua | *Ficus* sp. | TARI |
| CH2009FB14 | 2009 | Changhua | *Delonix regia* | TARI |
| CH2009MR15 | 2009 | Changhua | *Bauhinia variegata* | TARI |
| CH2009MR16 | 2009 | Changhua | *Ficus* sp. | TARI |
| CH2009MR17 | 2009 | Changhua | *Ficus* sp. | TARI |
| CH2009OL18 | 2009 | Changhua | *Ficus* sp. | TARI |
| CH2009MR19 | 2009 | Changhua | *Osmanthus fragrans* | TARI |
| CH2009LR20 | 2009 | Changhua | *Ficus* sp. | TARI |
| CH2009MR21 | 2009 | Changhua | *Cinnamomum camphora* | TARI |
| CH2009BG22 | 2009 | Changhua | *Ficus* sp. | TARI |
| CH2009FB23 | 2009 | Changhua | *Spathodea campanulata* | TARI |
| CH2009MR24 | 2009 | Changhua | *Pterocarpus indicus* | TARI |
| CH2009LR25 | 2009 | Changhua | *Ficus* sp. | TARI |
| CH2009MR26 | 2009 | Changhua | *Cinnamomum camphora* | TARI |
| CH2009LR27 | 2009 | Changhua | *Ficus* sp. | TARI |
| CH2010MR28 | 2010 | Changhua | *Ficus* sp. | TARI |

^a^ Each isolate is coded as its city/county of origin, collection year, host family, and serial ID. The host trees belong to 34 families (represented using 2-letter codes) including Anacardiaceae (AN), Annonaceae (AO), Palmae (Arecaceae) (AR), Araucariaceae (AT), Bignoniaceae (BG), Bombacaceae (BM), Combretaceae (CM), Cupressaceae (CP), Casuarinaceae (CS), Elaeocarpaceae (EL), Euphorbiaceae (EP), Fabaceae (FB), Fagaceae (FG), Hamamelidaceae (HM), Lauraceae (LR), Lythraceae (LY), Meliaceae (ML), Moraceae (MR), Myrtaceae (MY), Oleaceae (OL), Oxalidaceae (OX), Podocarpaceae (PD), Proteaceae (PT), Ericaceae (Rhododeae) (RH), Rhamnaceae (RM), Rosaceae (RS), Rutaceae (RT), Sapindaceae (SP), Sterculiaceae (ST), Theaceae (TH), Taxodlaceae (TX), Ulmaceae (UL), Vitaceae (VT), and unknown (UNK).

^b^ N/A: Host of some isolates were not identified or recorded by the collectors.

^c^ Isolate collectors and suppliers are indicated as follows: TARI, P. J. Ann and J. N. Tsai, Taiwan agricultural research institute; TFRI, T. T. Chang, Taiwan forestry research institute; TTDARES, H. L. Li, Taitung district agricultural research and extension station; NTU, collected by Y. C. Huang, Department of plant pathology and microbiology, National Taiwan university.
