# Supplementary material for: The Genetic Structure of Phellinus noxius and Dissemination Pattern of Brown Root Rot Disease in Taiwan
Source: PLoS One. 2015 Oct 20;10(10):e0139445. doi: 10.1371/journal.pone.0139445 (PMC4615629; doi:10.1371/journal.pone.0139445)
Supplement: S3 Table — TP: Taipei; HM: Hsinchu-Miaoli Hills, CW: Central West, SW: Southern West, YL: Yilan, and WV: East Rift Valley. (DOCX) [file pone.0139445.s005.docx]

**S3 Table. Pairwise *F*_ST_ values between the six geographical subpopulations**. TP: Taipei; HM: HsinChu-Miaoli Hills, CW: Central West, SW: Southern West, YL: Yilan, and WV: East Rift Valley.

|  | **TP** | | **HM** | | | **CW** | | **SW** | | **YL** | | **EV** |
| --- | --- | --- | --- | --- | --- | --- | --- | --- | --- | --- | --- | --- |
| **TP** | – |  | | |  |  |  |  |  |  |  |  |
| **HM** | 0.009 | – | | |  |  |  |  |  |  |  |  |
| **CW** | 0.0045 | 0.005 | | |  |  |  |  |  |  |  |  |
| **SW** | 0.0116 | | | 0.0025 | | | 0.0076 | | – | |  |  |
| **YL** | 0.0056 | | 0.0068 | | | -0.0012 | | 0.0064 | | – | |  |
| **EV** | 0.0126 | | 0.006 | | | 0.0066 | | 0.0038 | | 0.001 | | – |
