# Supplementary material for: The Genetic Structure of Phellinus noxius and Dissemination Pattern of Brown Root Rot Disease in Taiwan
Source: PLoS One. 2015 Oct 20;10(10):e0139445. doi: 10.1371/journal.pone.0139445 (PMC4615629; doi:10.1371/journal.pone.0139445)
Supplement: S4 Table — (DOCX) [file pone.0139445.s006.docx]

**S4 Table. Results of the analysis of molecular variance (AMOVA) assessing the proportions of genetic variations explained by the effects of geographical subpopulations, families of host trees, and collection years/time periods.**

|  | Whole population | | | |  | Taipei subpopulation |
| --- | --- | --- | --- | --- | --- | --- |
|  | Geographical subpopulations | Host families | Collection years | Collection time periods ^a^ |  | Genetic clusters |
| Percentage of variation among groups | 1.05% | 3.95% | 2.38% | 0.29% |  | 17.62% |
| Percentage of variation  within groups | 98.95% | 96.05% | 97.62% | 99.71% |  | 82.38% |
| Fixation index (*F*_ST_) | 0.0105 | 0.0395 | 0.0238 | 0.0028 |  | 0.1762 |

^a^ The collection years were divided into three time periods, 1989-1999, 2001-2009, and 2010-2012.
